# Supplementary material for: Public health risk communication through the lens of a quarantined community: Insights from a coronavirus hotspot in Germany
Source: PLoS One. 2023 Oct 12;18(10):e0292248. doi: 10.1371/journal.pone.0292248 (PMC10569635; doi:10.1371/journal.pone.0292248)
Supplement: S1 File — (PDF) [file pone.0292248.s001.pdf]

QUESTIONNAIRE

RISK COMMUNICATION |

The role of risk communication in pandemic management in the analysis of the outbreak in Neustadt am Rennsteig, Germany

PANDEMIC MANAGEMENT RESEARCH GROUP
11 May 2020

PD Dr. med. Dr. phil. **Petra Dickmann** MA

Clinic for Anaesthesia and Intensive Care Medicine (KAI)

Head and coordination of the

Pandemic Management Research Group

University Hospital Jena (UKJ)

petra.dickmann@med.uni-jena.de

The research was conducted in German. The material was automatically translated into English to allow international colleagues to access our data.

QUESTIONNAIRE RISK COMMUNICATION IN PANDEMIC MANAGEMENT - CASE STUDY THE OUTBREAK AND THE QUARANTINE IN NEUSTADT AM RENN- STEIG

PD Dr Dr Petra Dickmann, Head Dr Wibke Wetzker, Post-Doc

cand. med. Juliane Scholz, Stud. Assistant and doctoral

student cand. med. Annika Licht, Stud. Assistant

Please only fill in the questionnaire if you are over 18 years old.

QUESTIONNAIRE

I. General information

1. Age:_ _ years

2. Gender

□ male
□ female
□ diverse

3. How many people live in your household?

□ single
□ more than 1 person

II. Information

4. Which media or persons did you use to inform yourself about the coronavirus pandemic in Neustadt **before** the domestic quarantine was ordered (until 22 March 2020)? (Please tick a value between 1 and 5)

□ Daily newspapers

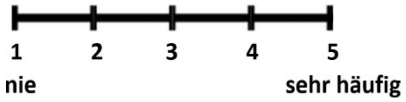

□ Television

1 2 3 4 5
nie sehr häufig

□ Radio

1 2 3 4 5
nie sehr häufig

□ Internet (general)

1 2 3 4 5
nie sehr häufig

□ Official authorities (online, e.g. RKI, BZgA, WHO etc.)

1 2 3 4 5
nie sehr häufig

□ Social media

1 2 3 4 5
nie sehr häufig

□ My life partner

1 2 3 4 5
nie sehr häufig

□ Doctor

1 2 3 4 5
nie sehr häufig

□ Pharmacy or other medical personnel

1 2 3 4 5
nie sehr häufig

□ Neighbours

1 2 3 4 5
nie sehr häufig

□ Other

5. Which media or persons did you use **during the** domestic quarantine (from 22 March 2020) in Neustadt to inform yourself about the coronavirus pandemic? (Please tick value between 1 and 5)

□ Daily newspapers

1 2 3 4 5
nie sehr häufig

□ Television

1 2 3 4 5
nie sehr häufig

□ Radio

1 2 3 4 5
nie sehr häufig

□ Internet (general)

1 2 3 4 5
nie sehr häufig

□ Official authorities (online, e.g. RKI, BZgA, WHO etc.)

1 2 3 4 5
nie sehr häufig

□ Social media

1 2 3 4 5
nie sehr häufig

□ My life partner

1 2 3 4 5
nie sehr häufig

□ Doctor

1 2 3 4 5
nie sehr häufig

□ Pharmacy or other medical personnel

1 2 3 4 5
nie sehr häufig

□ Neighbours

1 2 3 4 5
nie sehr häufig

□ Other

6. How did you receive information from the local authorities **during the quarantine**? (Please tick value between 1 and 5)

□ Internet

1 2 3 4 5
nie sehr häufig

□ Flyer

1 2 3 4 5
nie sehr häufig

□ Radio

1 2 3 4 5
nie sehr häufig

□ Home visits

1 2 3 4 5
nie sehr häufig

□ Announcements

1 2 3 4 5
nie sehr häufig

□ Other

7. How well informed did you feel **<u>during the quarantine</u>**? (Please tick a value between 1 and 5)

1 2 3 4 5
gar nicht gut sehr gut

□ There was far too much information

8. What information would you have liked to have **<u>during quarantine</u>**?

9. How worried were you **<u>during the quarantine</u>**? (Please tick between 1 and 5)

1 2 3 4 5
gar nicht sehr

10. What have you been worried about? (Please tick value between 1 and 5) My own

health

1 2 3 4 5
gar nicht sehr

The health of my family

1 2 3 4 5
gar nicht sehr

My mental wellbeing

1 2 3 4 5
gar nicht sehr

The economic stability of my country (recession)

1 2 3 4 5
gar nicht sehr

My personal financial situation

1 2 3 4 5
gar nicht sehr

The political stability of my country

1 2 3 4 5
gar nicht sehr

My job security

1 2 3 4 5
gar nicht sehr

Other

II. communication with the authorities

11. Which possibilities of contact with the authorities have you used? (Please tick a value between 1 and 5)

□ Telephone hotline

1 2 3 4 5
nie sehr häufig

□ Telephone appointment

1 2 3 4 5
nie sehr häufig

□ Online chat

1 2 3 4 5
nie sehr häufig

□ Other □

None

III. coordination

12. Did you find the quarantine appropriate to the situation?

□ Yes □ No

13. In your opinion, what proportion of Neustadt residents complied with the quarantine? (Please tick value between 1 and 5)

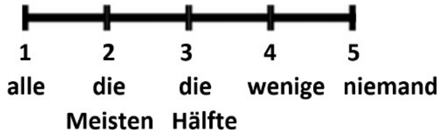

14. Can you understand why some people did not comply with the quarantine?

□ Yes

□ No

If so, why?

15. Did you rent certain places or groups of people after the end of the quarantine because you assumed a high potential for infection?

□ Yes

□ No

If yes, which ones:

16. If in other regions a domestic quarantine is ordered for a whole community or city, what measures do you recommend to the local authorities:

1.

2.

3.

Analysis of the open questions of the questionnaire on risk communication in Neustadt am Rennsteig according to Mayring's method

1. Determination of the material

   The material studied was taken from the risk communication questionnaire prepared by the Pandemic Management Research Group under the direction of Dr. Dr. Petra Dickmann on 11 May 2020 and answered by the residents of Neustadt am Rennsteig.
   This is an analysis of the open questions that appeared in this questionnaire. These questions are:

   Question 6.6 "How did you receive information during quarantine from local authorities?"
   — "Other:"

   Question 8 : " What information would you have liked to have during quarantine?" Question

   10.8 "What were you worried about?"
   — " Other"

   Question 14.2 "Can you understand why some people did not comply with the quarantine?"
   — "If so, why?"

   Question 15.2 "After the end of quarantine, did you avoid certain places or groups of people because you assumed a high potential for infection?" - "If so, which one?"

   Question 16 "If in other regions a domestic quarantine is ordered for the whole municipality or city, what measures do you recommend the local authorities to take?"

2. Analysis of the situation of origin

After a local accumulation of Corona infections in the town of Neustadt am Rennsteig, a quarantine was declared there at about 10 pm. at about 10 p.m., a quarantine was declared with loudspeaker trucks. This succeeded in containing and controlling the outbreak. At the end of the quarantine period, serological tests were carried out here in conjunction with questionnaires on various aspects of the quarantine.
This questionnaire was completed as part of this investigation.

3. Formal characterisation of the material

Written answers are given to open questions, although this questionnaire was already preceded by another one. This might have reduced the motivation to fill in the free fields.

4. Determination of the direction of analysis

The contents are divided into thematic areas or proposals according to their wording.

5. Theoretical differentiation of the question

How has the municipality's communication contributed to compliance or how can communication be improved with regard to a quarantine situation?

6. Determination of the analytical technique

In the following, the entries are structurally analysed and coded.

7. Definition of the units of analysis

The entries are classified thematically according to their content. Each theme is assigned a number here e.g. "1". Explicit demands or terms are then coded with a second number, e.g. "1**.2**". Each question has its own legend.

8. Carrying out the material analysis

Analysis of results for question 6.6 "How did you receive information during quarantine from local authorities?" — "Other:"

Results:

Statement (questionnaire number)

- Television, telephone (232)
- Information from the population itself, e.g. that a person who tested positive was shopping at the local supermarket wearing a mouth guard (91)
- WhatsApp from friends, family, phone (104)
- My family has always kept me informed (122)
- Whats up (127)
- Newspaper (133)
- Daily newspaper (138)
- Television (143)
- Phone calls (158)
- By word of mouth (151)
- Unfortunately, very little information! Also at the end of the quarantine no further information how it should go on afterwards! (149)
- Whatsapp (174)
- WhatsApp chat of the fire brigade (175)
- WhatsApp notifications to local residents among themselves (207)
- By written notification (226)
- Facebook (230)
- Not even our mayor had a few words for us. Sad!!!! (171)

In addition, 2 persons (119, 179) ticked the box " Other" without specifying it below.

1. Selection

In addition to the options given in question 6 (internet, leaflets, radio, home visits, announcements), the following sources of information were also mentioned:

- WhatsApp (104, 127, 174, 175, 207)
- Television (143, 232)
- Telephone (232, 158)
- Family (104, 122)
- Friends (104)
- Newspaper (133,138)
- Information from the population itself (91)
- Word of mouth (151)
- Facebook (230)
- Written notification (226)
- Carer (285)

And there are two more entries that are more in the nature of comments and thus do not serve to evaluate this question.

"Unfortunately, very little information! Even at the end of the quarantine, no further information on what happens afterwards.

should go!" (149)

"Not even our mayor had a few words for us. Sad!!!" (171)

Thematic summary and coding:

The results were not summarised according to their medium, as this is difficult with the frequently mentioned medium "WhatsApp", for example. The addressees of such information are usually acquaintances/relatives, but the prerequisite for receiving this information is both access to the internet (it is a Facebook company), but also the possession of a mobile phone number. Thus, it can neither be clearly assigned to the category "internet" nor to the category "telephone". It would also be incorrect to group it with the parent company and term "Facebook", since all that is needed is internet access.

Since this questionnaire focuses on risk communication, i.e. the flow of information, the terms "WhatsApp", "family", "friends", "word-of-mouth" and "information by the population itself" can be grouped together with the term "telephone", since the latter is a preferred medium among those involved (i.e. relatives and acquaintances). If we consider the population of the village as one level, there is a horizontal flow of information.

A vertical flow of information (i.e. a flow of information from the level of decision-makers to the level of the population) can take place via the newspaper or other written notifications, the internet, announcements, and also television.
However, since each of these media can also provide a flow of information of a horizontal nature (experience reports, etc.) and do not necessarily bring new information with them, all these media are coded individually.

**Category**	**Topic**	**Anchor term**	**Definition of the anchor term**	**Coding**	**Participant**
1. Vertical communication	Newspaper	Newspaper	"Newspaper" (133)	**1**	**133,138**
	Written Notification	Written Notification	"Written Notification" (226)	**2**	**226**
	Television	Television	"Television" (143)	**3**	**143,232**
	Facebook	Internet	"Facebook" (230)	**4**	**230**
2. Horizontal communication	WhatsApp, telephone, friends, family, word-of-mouth, information through the population itself	Personal environment	"Word of mouth" (151)	**5**	**104,127, 174, 175, 207, 232, 158, 122, 91, 151,285**

Coding is done with letters and numbers to account for ticked but unspecified answers (these are coded "0").

Analysis of the results for question 8 : " What information would you have liked to have received during the quarantine?" Coding:

**Category**	**Anchor term**	**Definition of the anchor term**	**Coding**
**Course**	**Course of the quarantine**	"More detailed information about the course" (110)	**1**
**Tests**	**Tests**	"More detailed info, when and where Test until the first Fleyer, a lot of things had to be done by the Ask health department." (97)	**2**
**Epidemiology**	**Infected persons**	"More information about infected people in one's own environment, and on how to deal with them properly". (178)	**3**
**Supply**	**Supply**	"Supply of medicines, delivery of mail and parcels (deliveries only after 3-4 days regulated)" (149)	**4**

**Behaviour**	**Information on the correct behaviour**	"More detailed information on behaviour during quarantine" (94)	**5**
**Satisfaction**	**The information were sufficient**	"We felt well informed." (121)	**6**
**More Information**	**General more Information or a better Communication**	"Announcements were not understandable (residential location), information rather sparse" 148	**7**

Results:

Questionnaire	Statement	Coding
2	let us know earlier (002)	1
71	On the day the quarantine was lifted	1
91	I have asked several times if those who test positive are allowed to return after the<br>Standard 14 days another test is carried out. Unfortunately, no answer.	2
95	What happens next	1
94	More detailed information<br>on behaviour during quarantine (094)	5
97	More detailed information on when and where tests are carried out had to be obtained from the health department until the first Fleyer.	2
98	None	6
99	We felt well informed	6
101	All information was optimal	6
103	All information was optimal	6
102	All information was in order	6
104	In the beginning, I had to clarify myself whether I could take the dog for a walk - it was not clear, opening times of the sales facility	4, 5
105	I was in hospital at the time!	0
107	Reliable information on patients who have already recovered,<br>age structure of the Infected	3
108	Info was very good	6
109	Reliable information on patients who have already recovered,<br>age structure of the Infected	3
110	More detailed information about the course	1
111	The end of the quarantine was unclear to me because of the high number of infected people. However, it was announced at 10.15 p.m. that our town would be quarantined. We heard the end of the quarantine on the radio. There was no<br>Announcement about the fire brigade. That was not well managed at all.	1
112	All the information came to us through social media. Also the Announcements were mostly understandable.	7
113	More education and not feeling so alone.	7

114	I was sufficiently informed!	6
115	Organisational info	1, 4
116	None, everything was very good	6
118	Whether there is another test for those who test positive	2
119	That all federal states follow a uniform path.	0
121	We felt well informed.	6
127	Why was qurantine imposed, what happens after the 14 days are over?	1
128	General information	1
131	Information on what has happened and why the need exists	7
132	Meaningful info	0
139	Better Coodination Announcements Have Often Overlapped, Fleyer Though<br>good, but who looks in the letterbox when there is no mail delivery	4
140	What happens next.	1
141	How many infections, who	3
143	Was sufficient!	6
145	Clarification about what happened from the beginning	7
146	Everything was ok.	6
147	More announcements!!!, information is mostly only available via Facebook get<br>(very bad)	7
148	Announcements were not understandable (residential location), information rather sparse	7
149	Supply of medicines, delivery of mail and parcels<br>(Deliveries only settled after 3-4 days)	4
150	Finally, an announcement to everything as not all pensioners have internet or mobile phones!	1
151	Secure information through official bodies and not so many "Conjectures" of some media	7
152	Current situation	7
153	About the end of quarantine	1
156	More information from the mayor!	7
159	How is the place supplied? What about animal care?, who was positive?	4, 3
160	One had the impression that everything was completely disorganised.	1
161	Everything fine	6
164	Official case numbers of infected persons, further action in the first Week	1, 3

168	Faster information, better announcements (louder and better understandable)	7
170	Procedure (whether to test/when to test)	2
171	More info by the ...mayor	7
172	No message came from the mayor to the residents of our village	7
173	More info about our place and rules of conduct	5
175	**statements that authorities, in particula · the district administration, also partially t quickly and could never are overwhelmed because they had to ac rehearse the case beforehand**	7
178	More information about infected people your own environment, and on in the correct Dealing with it	3, 5
179	Throwaway in the letterbox.	7
181	everything was good	6
183	Better and more precise information also i ı the side streets	7
187	Information about current status	7
192	excellent(organised)	6
208	was well organised	6
214	State of affairs	0
220	The quarantine began on Sunday evening. One would have on Monday morning have to provide the residents information. (medication, c.) with meals on wheels, doctor's appointments, et	4
230	There was sufficient information	6
234	The quarantine began on Sunday evening. One would have on Monday morning have to provide the residents information. (medication, c.). with meals on wheels, doctor's appointments, et	1
243	It was enough, everything was ble understanda	6
250	Everything was sufficient	6
251	Everything was sufficient	6
253	None	6
254	General information from the mayor. But there was zero info	7
273	Why quarantine-without-testing ordered r healthy people fo was	2
274	Daily status of infected persons, online ne vs, punishment of persons who have carried out so-called "C ›rona parties".	3
277	To be addressed by the local mayor and di trict administrator	7
278	Official address by the mayor of the village	7

279	To be addressed by the local mayor and di trict administrator	7
286	Everything fine	6
287	Optimal	6
289	Announcements in all streets and at different times, clear line, not such a chaos	7

Analysis of results for question 10.8 "What were you worried about?" — " Other"

Results:
Answer (number of the questionnaire)

- Negative incidents during the quarantine were incomprehensible to me, annoying and damage to the image of the place made me angry (90)
- I was very worried about humanity itself. Through the media we saw terrible pictures from Italy, Spain, etc. (coffins, mass graves). I often asked myself whether and how the virus would change the world and the people. (97)
- None (98, 253)
- My family in the neighbouring town -> are care leavers (104)
- Social life (126)
- Normality and stability afterwards (149)
- As self-employed people, we have been responsible for at least 6 families for almost 30 years, securing jobs was our top priority (150)
- About an extension of the quarantine because a few people did not comply with the regulations and in some cases had parties with infected persons. (274)

Anchor example	Definition of the anchor example	Questionnaires	Coding
Worries about (vulnerable) People	"My family in the neighbouring village -> are care cases" (104)	**97, 104**	**1**
Worries about life afterwards (social, economic)	"Normality and stability afterwards" (149)	**149, 90, 126, 150, 274**	**2**
None	"None" (98)	**98, 253**	**3**

<u>Analysis of Question 14.2 "Can you understand why some people did not comply with the quarantine?"
— "If so, why?"</u>

Results:
Answer (number of the questionnaire)

- The supply in the village was well secured (92)
- There are stupid and stubborn people (93) - This was a new situation for all of us (95)
- Personal reasons more important, locked up too long, not convinced of the situation (104)
- **Lack of control, lack of information due to too many different info's from different sources (107)**
- Being a high-risk patient myself, one has a different view of violations (124)
- There are idiots everywhere (132)
- Family celebrations, birthday parties, etc.(142)
- Because it came so suddenly and no one knew what was happening right (how it would continue) (145)
- Earlier reaction would have reduced the extent (tests), overreaction by law enforcement, authorities (148)
- Insecurity and fear, indifference, risk, stupidity (149)
- Emergencies e.g. In the family (151)
- Because it goes against basic rights and is not done that way in other regions either (152)
- Little to no intelligence (167)
- Because they do not perceive the disease as a serious threat. (170)
- because they did not know any other way (181)
- there are always lateral thinkers (190)
- **It had the appearance of an experimental laboratory.(194)**
- Because there are always those (226)
- Death (243)
- Because they were afraid (275)
- Not enough information (277)
- Restriction of freedom (284)

**Category**	**Anchor term**	**Definition of the Anchor term**	**Coding**	**Questionnaires**
A intrinsic Reasons	**Lack of intellect**	"Little to no intelligence" (167)	**1**	**93, 132, 149, 167, 226**
	**Uncertainty**	"Because it was so sudden and nobody	**2**	**95, 107, 145, 149, 181,275, (287???)**
		knew, what happens right		
		(as it continues)" (145)		

	**Lack of understanding**	"Personal reasons more important, locked up too long, not convinced of the situation" (104).	**3**	**104, 148, 149, 152, 170, 190, 194, 284, 277**
B extrinisic reasons	**Societies**	"Family celebrations, Birthday parties, etc." (142)	**4**	**142**
	**Emergencies**	"Emergencies e.g. In the Family" (151)	**5**	**243, 151**

The statements of respondent 92 and respondent 124 could not be assigned thematically.
These responses were coded as " 0". The categories are not included in the coding here for simplicity, but can be taken into account in the analysis.

Analysis of the answers to question 15.2 "Did you avoid certain places or groups of people after the end of the quarantine because you assumed a high potential for infection?" - "If so, which one?"

**Questionnaire Statement**90	**Coding**10, 60

Shops, neighbours, own relatives (90).

56Nearby purchase (56). 11

Categorisation:

**Topic**	**Anchor example**	**Definition of the anchor term**	**Coding**
Business	Shopping market,	"Shopping centre" (244)	**10**
	-of which explicitly Nahkauf:	"Nahkauf" (56)	**11**
Hairdresser		"Hairdresser" (96)	**20**
Medical Facilities	Medical Facilities	"Medical practices" (200)	**30**
People	Risk patients	"elderly people (parents), grandchildren" (219)	**40**
	Foreign	"To all strangers<br>Kept at a distance" (151)	**50**
	Acquaintances and relatives	"Other relatives and friends" (253)	**60**
	Persons infected with SARS<br>COV2- were infected	"All who have had it." (133)	**70**
	Crowds		**80**
	People in general		**90**

Note: Statements such as "Gatherings", "General places" "Large gatherings of people" were assigned to group 3B "Strangers", as an occurrence of such persons cannot be foreseen here.

Results:

89Shop (89). 10

91	After the quarantine period had expired (positive test), people were seen coughing heavily in the local supermarket and in the town. were on the road. I avoided those people. (91).	70
96	Hairdresser, department store (96).	10,20
98	Large crowds, events 98)	80
100	Health (100)	0
101	Friends, neighbours, large crowds (101).	60,80
102	Friends, neighbours, large gatherings of people (102).	60,80
103	Large crowds, friends, family, all people where it was possible to avoid them (103)	60,80
105	No contact! (105).	90
106	No contact (106)	90
108	Contact with other people (108).	90
111	You don't feel attracted to any groups. I have been on In the beginning I only had contact with my family. (111).	80
114	Shopping centre, public transport (114).	10
115	My daughter with family, especially the grandchildren, as well as ill Brother (115)	60
118	Grandparents, large supermarkets (118).	60, 10
120	Didn't go to the hairdresser and didn't go shopping. Have my Granddaughter and great-granddaughter not seen. (129).	10, 20, 60
122	I stayed at home and my daughter did everything for me. Done (122)	90
124	Shopping centre (124).	10
125	Especially festivals and other events (125).	80
127	Nahkauf (127).	11
129	Risk areas, gathering of crowds, Prohibition of contact (129).	80, 90
130	Grandparents, transregional family members (130).	40, 60
131	shopping centre, as I am a high-risk patient (131).	10
133	All who have had it. (133).	70
134	Shopping facilities (134).	10
135	Distance (2m) (135).	90
136	Distance (2m) (136).	90
137	Pensioners (grandparents, great-grandparents) (137).	40
138	Crowds, our children, sports group (138).	80, 60
139	Larger retail chains (139).	10
140	Nahkauf (140)	11
141	All except shopping market (141).	90
142	groups that were infected (142).	70
143	Where there were many people! (143).	80
144	General measures (144).	0
146	Discount store, larger gathering of groups of people (146).	10, 80
147	My neighbour!, general locations (147).	60, 0

	All (160).	
151	Kept at a distance from all strangers (151)	50
153	Supermarkets in other towns, public buildings, doctors' surgeries (153).	10, 30
154	No shopping, no events, no crowds of people (154)	10, 80
155	No shopping, no meetings (155).	10, 80
157	Shopping facilities (157).	10
159	All groups of people, shopping centre (159).	10, 80
16090, 10		
161	Specialists (161).	30
162	Specialists (162).	30
165	Shopping, meetings, many people (165)	10, 80
168	Shopping malls, cities, events, contact with colleagues, clients, other institutions(168).	10, 60, 80
169	I generally avoid large groups of people since the beginning of the pandemic(169)	80
170	Shopping centres(170)	10
171	My grannies.(171)	40
175	Generally left children at home, only went shopping alone max. 1x per week in the evening! (175)	90
176	Supermarkets in other towns, doctors' surgeries (176)	10, 30
177	Doctors, building and supermarkets(177)	10, 30
178	Supermarkets, known infected persons, doctors' surgeries(178)	10,70 , 30
179	Neighbours! Mall! (179)	10, 60
189	in the wider family (189)	60
191	Supermarkets etc. (191)	10
192	HA. Quarantine; pers. Quarantine (192)	0
193	Department store(193)	10
195	Shopping(195)	10
198	Persons were positive (198)	70
200	People outside the family, large supermarkets, doctors' surgeries (200)	50, 10, 30
207	by the general situation as few contacts as possible e.g. when shopping (only 1x a week) (207)	90
212	Shopping market! Also during quarantine (212)	10
215	Nahkauf (215)	11
219	elderly (parents), grandchildren (219)	40, 60
221	as few contacts as possible, only 1x a week for shopping (221)	90
224	Crowds (224)	80

226	I was positive from5.04.20 (226)	0
229	Contacts with family, acquaintances, supermarkets outside the village (229)	60, 10
231	Shopping Hall (231)	10
234	Large supermarket, general shopping (234)	10
235	Coronavirus is still there (235)	0
236	Rewe, Nahkauf (236)	10
237	Parents, as they fall under risk group (237)	40
238	Local Rewe/ Nahkauf (238)	10
239	Purchasing (239)	10
243	To all strangers and to own family (Munich/ Eisfeld) (243)	50, 60
244	Shopping centre (244)	10
252	Shops (252)	10
253	Other relatives and friends (253)	60
256	Neighbour, Family (256)	60
258	Elderly people or with pre-existing conditions (258)	40
261	Larger department stores (261)	10
262	Shopping markets, events (262)	10,80
263	Have little contact (263)	90
264	Several persons (264)	90
269	All (269)	90, 10
272	Where high contact was possible (272)	80
278	Department store, family, friends	10, 60
279	Department store, family, friends	10, 60
284	Family, friends, school	60
286	All	90, 10
287	All	90, 10
289	Nahkauf	11

Question 16 "If in other regions a domestic quarantine is ordered for the whole municipality or city, what measures do you recommend the local authorities to take?"

The classification and coding is done thematically.
Similar statements were summarised here (see anchor examples and definitions), whereby it was not always clear which category or coding the statement could be assigned to. Also, several points were often mentioned in one sentence.
In such a case, this sentence was either split and the parts coded respectively or multiple codings were made for one statement.

Category	Anchor example	Definition of the Anchor example	Coding
1<br>Statement from Satisfaction, recommend simultaneous measures	"New Town Model	Implementation of simultaneous measures as they were taken in Neustadt or the communication carried out there<br><br>"Do exactly as here" (108)	11
2<br>Tests	Previous tests	"Faster test" (93)	21
	More tests	"Faster tests too with no symptoms" (172)	22
	Good organisation of the Tests	"Good organisation in the Test management" (116)	23
3<br>Controls	(More) Controls	"Controls whether ordered quarantine is observed." (91)	31
	Punishment from Misconduct	"In case of noncompliance this should be punished, that's what I expect from the Government!" (143)	32
4<br>Supply	Secure and organise supply	"good supply of food of the Residents" (211)	41
	Medical care	"Medical care." (196)	42
5<br>Rules restricting public life	None<br>Allow major events to take place	" None<br>Major events" (98)	51
	Allow walks,<br>Permitting condolence visits	"Permit from Walks (alone) with plenty of distance in the vicinity (field and forest) in the community" (100)	52

	Keep distance	"Keep your distance" (98)	53
	Mandatory Mouth-Nose Protection	"Wearing Masks" (265)	54

	Stricter quarantine	"Families with ll<br>infected relatives atine<br>under strict quaranrect<br>and not only the di<br>infected persons".<br>(178)	55
6<br>Information	On the course of the Quarantine	"Structured info e.g.<br>Rubbish collection, mail<br>and parcel receipt<br>before quarantine rders<br>o / Emergency n<br>plan i place be"<br>(118)	61
	No misinformation or sensationalism	"For residents, no ıe<br>untrue reports on<br>t internet publish"<br>(90)	62
	Earlier announcement of the<br>Quarantine	"Quarantine befor<br>announce(...)" (166	63
	More information	the number of infected<br>people, but also<br>the general<br>demand	64
	Dissatisfaction with the way the quarantine start (and - end) is announced (proclamation).	"Quarantine start as<br>w announced after nset<br>su with blue lights d<br>an sirens and ker<br>loudspe r<br>announcements- s was<br>fo children (7+10)<br>thi very dramatic<br>and frightening"<br>(176)	65
	Wide range of<br>Use media (digital, analogue and personal)	"Since it is difficult to<br>find a<br>common medium<br>should be used ers,<br>(fly social "<br>media...),<br>Announcements...)<br>(130)	66
	General more information	"Always inform people<br>about everything s) erly<br>that everything<br>happens in an ord<br>and calm manner.<br>goes." (119)	60
7 Criticism Leadership	Comments that the	96) Support for old<br>and sick people by	7

	Explicitly concern leadership	community members -> ask (Mayor)	

If you put the codes and the answers together, you get the following picture:

Testarc 1. proposal

**56**	(56) control more,
**88**	(88) Caution (?)
**90**	(90) Do not publish untrue reports on the internet for residents.
**91**	(91) Controls whether ordered quarantine is observed. Was introduced much too late in Neustadt
**92**	Earlier mass tests, was much too late in Neustadt introduced
**93**	93) Faster test,
**94**	94) Better planning, better information,
**95**	95)More information for concerned citizens
**96**	96) Support of old and sick people by community members -> ask (mayor)
**97**	97) Earlier information and no "night and fog" actoin
**98**	98) Keep your distance
**99**	99) In the case of necessary measures, no attention can be paid to any "urge for freedom".
**100**	100) Allowing walks (alone) with plenty of distance nearby (field and forest) in the Community
**101**	101)The same as in Neustadt
	102)The same as in

			Coding	Proposal 2	Coding	Proposal 3	Coding
			31	further test after 2 weeks (in case of positive test)	22		62
**102**	Neustadt	11	53				
			62	Strict controls	31	truthful reporting in the press	
			31				
			21				
			21	Separate healthy and sick	0		
			63	Not so late in the evening so that you have time to organise everything, e.g. inform your employer.	65		
			64		0		
				Less fire brigade			
			7		41	Mandatory mouth	
				Maintaining shopping facilities			
							54
			65	**Better information flow about numbers, future tests etc. so that people do not feel let down**	64		
			53		51		
				No major events			
			0				
			52	Supply through local shopping was guaranteed. In places without Sales facilities	41		
			11	It is essential to create opportunities for supply (daily needs)	41		

**103**	103)The same as in Neustadt!	11				
**104**	104)Sufficient information: daily status	64<br>64	Daily announcement of the Infected persons (number of cases)	64		
**105**	105)Calls, announcements		Behavioural sailing	61		
**106**	106)Communication with citizens (calls, rules of conduct, etc.)	66<br>31	Public medical measures	42		
**107**	107)Concrete controls	11	More precise measures	61	Accessibility	7
**108**	108)Do exactly as here					
**109**	109)Better information on the course of the disease of infected persons and the number of new infections	64<br>64				
**110**	110)Better information for citizens	60	More detailed information on contagion figures	64		
**111**	111)Proper communication					
**112**	112)Good information for citizens, older people do not have social media	66<br>64	Supply of the citizens, perhaps at least Asking if other people provide them with food supply	41,7	Better information to the staff of the health department , had to call daily, but for specific questions they had to ask first themselves as they had no info. They really tried hard, were also very friendly and helpful, their job would be made easier if they had received more info.	64
**113**	113)More for the citizens with Do enlightenment					
**115**	115)Citizens must be comprehensively informed, public announcements are not Sufficient	64<br>11				
**116**	116) The New Town Model	11	More and stricter controls with fewer Talkability	31<br>32	Good organisation in the test management	23

117	(117)Several controls and information as in Neustadt		Strict penalties for violations		guaranteed the supply of the inhabitants,	41
					Do not forget elderly and sick citizens as it worked out in Neustadt	
118	(118) Structured info e.g. waste collection, post and parcel<br>Receive before quarantine orders / emergency plan in place	61	Information dissemination to older citizens without internet.	66	0	
119	(119)Always inform people about everything so that everything is done in an orderly and calm manner.	64	0		0	
120	(120)Good information	60	0		0	
122	(122)More aid measures	41	0		0	
123	(123)Announcement of quarantine too late (time). Employer had to be informed	65	Opening of Neustadt was not enough announced.	65	0	
124	(124)Timely information on the current situation	60	0		0	
125	(125)Information several times a day about the current Situation	61	Immediate curfew	54	0	
126	(126)Home testing (door-to-door)	23	Clarify how people who are in domestic quarantine in addition to local quarantine get food.	41	Condolence visits - family & inner city- allow	52
127	(127) Clarify beforehand how everything can and should proceed.	61<br>64	How do I regulate the Safeguarding the volunteer helpers	61	0	
128	(128) More detailed information	11	0		0	
129	(129) Like distributing flyers in Neustadt, giving info		Loudspeaker announcement n: clear prohibitions pronounce	64	0	
130	(130) Citizens should be better informed (more often).	64	As it is difficult to find a common medium, all should be used (flyers, social media, announcements...).	66	0	
131	(131) Check whether a quarantine is necessary for an entire locality.	0<br>41	Mass test at the beginning so that the entire place is not under must be placed in	21<br>31	70	

		7	quarantine	52		
**132**	(132)Enough volunteers,		Controls		0	
**133**	**(133)Stay calm the** **Give people the feeling of security,**		still let me go for a walk but alone.		0	

**134**	(134)Carry out controls	31	0		0	
**135**	(135) Restriction of freedom of movement	53	No major contact to other persons	53	Coping with w h a t t h e authorities dictate	0
**136**	(136) Restriction of freedom of movement	53	No major contacts with others People	53	Coping with w h a t t h e authorities dictate	0
**137**	(137) Do not drive through the village at 9 pm with the fire brigade and make the injunction.		0		0	
**138**	announcements.<br>(138) Regular Controls,	65<br>31	Establishing a local telephone number that anyone can reach at any time, when he needs help	7	0	
**139**	(139) Exercises, such as for railway tunnels/motorway tunnels Fire drills for specific fire incidents etc.	61	0		0	
**140**	(140) More controls	31	At the end, another test. The positive person tested. Virus does not disappear abruptly after 2 Weeks.	22	0	
**141**	(141) Info	60	0		0	
**143**	(143) It is a social responsibility that the quarantine rules are respected. for the people	31	In case of non-compliance, this should be punished, this is what I expect from the Government!	32	0	
**144**	(144) Waste disposal,	41	Hiking trails Labelling,	52	Quarantine	11
**145**	(145) Prior clarification and exact course description of the Quarantine	61	0		0	
**146**	The current designation of the infected (via the Data protection beyond)	**64**				
**147**	(147) Frequent control by the police	31	In case of violation high Fines	32	Much better clarifications and ORDINARY Announcements!!!	64
**148**	(148) Is it really the last resort? Or is domestic quarantine enough	64	The supply of mail and medicines was good, Food	41		

149	(149) More information and preparation time	61, 64	All supplies should first be clarified before closing (medicine), Food)	41		0
151	(151)Secure information 62		Review of the measures and the Possibility to ensure exceptions (Family emergencies)	52	After that, perhaps some praise to the residents who have shown exemplary commitment from "top" position.	7
156	(156) Should seek advice from our local authorities 11			0		0
157	(157) Talking to all citizens more to understand why, especially for older citizens, would be very important.	7		0		0
158	(158) Info-ok-Safety-Tolerance Protection-Self-protection hyped up by media	0	Info phone for questions - not quarantine-concerning	7		0
159	(159) Proper and appropriate education and not just cars with loudspeaker announcements	65 + 64	Before clarification of how the supply should work	61		0
160	(160) Clarification of all necessities such as supply	41	More communication	64	Where was the politics? The place shut down and good. That was not in order and caused great insecurity. diffused.	7
161	(161) Don't seal off the transport when all possible entrances and exits of the place have to be closed. More police officers should be available stand!	31		0		0
162	(162) Residents better inform	64	Alone walks allow	52	More thorough disinfection of local shopping facilities (Hygiene!!!)	41
163	(163) Don't ban everything.	5->52	Prepare better (coordination of emergency forces and Logistics)	41 + 61	Residents better test and inform	21
164	not at the end.	21	Inform citizens well further procedure	61	Announce quarantine in advance so that each individual can follow important procedures e.g. Shopping, Bank,	63

	beginning of the quarantine,		about the process and			
					pharmacy, etc., and not just "close" on a Sunday evening without warning. make	
**166**	(166)Giving businesses in the locality the time to develop their Organise business!!!	63	Inform all residents. Not first after 3 days.	63	0	
**167**	(167)In case of noncompliance high fines! -> Act more quickly in the event of charges!	32	MORE controls by the police	31	Better information through say!	64
**168**	(168)Frequent control of compliance with quarantine	31	Faster response from authorities when citizens notice and report non-compliance	32	High fines	32
**169**	(169)Ensures the supply	41	Organises the delivery of the mail	41	0	
**170**	(170)Communicate clearly WHY a quarantine is ordered (case numbers of possible scenarios WITHOUT quarantine).	64	(		(	
**171**	(171)More info by the mayor More info from the Health Department	7	Faster tests even if there are no symptoms	22		
**172**	(172)Better education of the residents	64	The Mayor must become much more endeavour to inform what happens with us Mrs Enders and M. Hettstedt took over.	7	More clarification about the situation, what, when, who does	61
**173**	(173)Better info to the residents	64	Not to be observed like that.	0	Better communication of the Mayor, who is at all not about us has taken care of.	7

174	(174)Sufficient protective masks, disinfectant, Protection notices etc.	41	Emergency plan for public life, such as provision of medicines, daily necessities, medical care, etc. Supply	61+ 41	An information channel/platform where all citizens get the same information base to avoid FakeNews. Block info channels with false reports.	62
175	(175)Open communication-> demand understanding, openly communicate that arrangements for care etc. are still come, but are currently being worked out!	7	Simply omit disparaging remarks by the district administrator in the media! Interview, how could this happen! Cofidis-19 developed rapidly, at the beginning of March there were still many people all over Germany at family celebrations!	7	! Functioning mail and parcel service helped a lot to survive the quarantine!	41
176	(176)The start of the quarantine was announced after sunset with blue lights and sirens and loudspeaker announcements - for children (7+10) this was very dramatic and frightening.	65	Conduct mass testing in a more structured way - like here by letter or by street - the instructions on who, if and when to test were very confusing and partly contradictory.	23	Information on the Q. Make better digitally accessible e.g. through the LRA homepage	66
177	(177)The timing of the announcement of the "Q" was inconvenient Children 7+10 were afraid0	65	0			
178	(178)Families with infected relatives all under strict quarantine and not only the direct infected persons.	55 64	Test the whole community immediately and not after several days	21	Better Care for single or elderly persons via mobile care which are centrally organised and set in motion becomes	41
179	(179)Better information to the citizens about the first measures!!!		0		0	
180	(180)Faster information on the expected duration, type of supply option (food, etc.),	64 21	Provide medical staff	41	direct contacts name	7
182	(182)faster testing	61 + 65	0		0	

**183**	(183)better planning, Better information (no Night and fog action)		0		0	
**184**	(184)Blue light and loudspeaker announcement is already frightening, but safe appropriate	65<br>11	0		0	
**185**	(185)just like in Neustadt - proper information about the actual Conditions		0		0	
**186**	(186)more education about the situation; more planning	61 + 64	0		0	
**188**	(188) good supply;	41	Inform properly	64	0	
**191**	(191)none - everything was good that way	11	0		0	
**196**	(196)Quarantine makes sense.	->>>11<br>11<br>64	Medical care.	41	Control of the measures very valuable.	31
**197**	(197) Everything was fine.	31	0	32	0	
**198**	(198)much enlightenment		0		0	
**199**	(199)steady control		and severe penalties		0	
**201**	(201)The quarantine does not to impose in the middle of the night! One has found oneself in a war-like state! One has found oneself very felt leprous.	65<br>31	0		0	
**202**	(202)Checking the shopping trolley (disinfection)		0		0	
**203**	(203)The authorities must have an opinion and also just give an instruction. It is better to consult the offices with those responsible on the ground.	7<br>31<br>31	0		0	
**204**	(204)should abide by the rules	64 + 63	0		0	
**205**	(205)Stricter controls		0		0	
**206**	(206)better + faster Information for citizens	61	0		0	
**207**	(207)precise information on Length (duration) of the Quarantine	65	0		0	
**208**	(208)Announcements were Hard to understand		0		0	
**209**	(209)better information dissemination of the authorities to those affected;	64	tougher penalties for violations	32	0	

210	(210)Announcement of quarantine not at late hour (10pm) as some did not hear the announcements.		0	0
211	(211)good supply of food for the residents	65<br>41<br>63<br>64<br>64	0	0
213	(213)timely info		0	0
214	(214)more information		0	0
215	(215)Sufficient information to data subjects or resident		0	0

66	0		0	
64<br>64	0		0	
61	0		0	
	0		0	
7<br>11	0		0	
11<br>11	0		0	
	0		0	
11	0		0	
31	More controls should be carried out		0	
21<br>11	0		0	
0<br>0	0		0	
11	0		0	
11	0		0	
31 + 55	0	31	0	

**216**	(216)Regular information through flyers and announcements, internet and TV	0	0
**217**	(217)approach the matter with leisure and try to inform the population even better; simply locking away is not the ideal way	0<br>0	0<br>0
**218**	(218)Information vis-à-vis the citizens		
**223**	(223))simple, better Coordination		
**225**	(225)Extensive information, also during the quarantine period; involve medical institutions/associations to cope with the quarantine period; pay particular attention to single and elderly people; deal calmly and objectively with the existing situation; prevent possible "bullying" via social networks; respond to people's fears/needs. enter		
**227**	(227)Measures as in New town		
**228**	(228)Same measures as in Neustadt		
**229**	(229)As in Neustadt		
	(230)the measures were fin and can be carried out in the same way in other **230** municipalities.		
	(232)That they strictly enforce, that the quarantine **232** is respected		
**233**	(233)First test then quarantine		
**235**	(235)The same as in Neustadt		
**237**	(237)Keep calm and do not panic!		
**239**	(239)Ask for advice		
**240**	(240)As in Neustadt		
**241**	(241)Measures see Neustadt /Rennsteig		
**242**	(242)Crackdown, let everyone comply with quarantine		

**243**	(243)Consistently monitor all adopted measures!	31		0	0
**245**	(245)More should be done to talk to people to remove hardships and at the same time educate them. operate.	7		0	0
**246**	(246)Do not inform residents late at night,	65	but days in advance for possible blocking inform.	63	0
**247**	(247)The same Measures like those taken for our place.	11		0	0
**248**	(248)It must be ensured that sensational journalism is avoided in any case (in the sense of BILD and Co.).	62		0	0
**249**	(249)Information from the Citizens	60		0	0
**250**	(250)Follow	31		0	0
**251**	(251)To follow	31		0	0
**252**	(252)Better info to community	64	Better communication and Organisation	61	0
**253**	(253)Further measures (e.g. Hazardous waste)	41		0	0
**254**	(254)Better and more comprehensive information	60	Faster Support and supply	41	0
**255**	(255)Ensure strict compliance!	31		0	0
**257**	(257)Mass testing not on a voluntary basis! Door-to-door testing and not on a place with tents	23		0	0
**259**	(259)Stronger crackdown and tougher, more consistent punishment and publication	32		0	0
**265**	(265)Wearing masks,	54	Keep distance 2m	53	0
**266**	(266)Generally existing orders sufficient	11		0	0
**267**	(267)Timely announcement of the quarantine (in Neustadt we were informed at 10 p.m., which was was spooky)	65		0	0
**268**	(268)Better education/information of the population	64			0

269	(269)More detailed COMMuniCation	64	Monitoring of important social media channels	31	0	
270	(270)Fast, orderly and constant information of the residents	64	0		0	
271	(271)Good and fast info	64	0		0	
273	(273)Possibly carry out the mass test immediately afterwards so that healthy people are not locked up for 14 days.<br>are	21	Also older people who have no internet, mobile phone etc. can be informed personally. Provide personal information, loudspeakers of the fire brigades were poorly audible or not audible at all.	66	0	
274	(274)Better announcements, for all roads in the municipality from the police, fire brigade (General more Information).	64	Better organisation by the authorities/municipality, e.g. Covid 19 tests.	23	Better supply of food. Each person/household would have to have a standardised selection and quantity of food delivered to their respective residences, so that there is no risk of infection in the local shopping centre arise.	
275			better info. The population		0	
277	(275) Earlier (and better info). The population	63	0	64	0	
	Better interaction with citizens					41

	Better information	64				
	Test earlier	63	More sensitive info for older citizens	64		
	The same as in Neustadt	11				
	Faster and more consistent decisions	61	Use police for protection	0	Severe penalties for violations	32
	Faster and more consistent decisions	61	Use police for protection	0	Severe penalties for violations	32
278	[illegible]etter [illegible]rmation	7				
		64				

279

18

282

284

286

287
