# Supplementary material for: Public health risk communication through the lens of a quarantined community: Insights from a coronavirus hotspot in Germany
Source: PLoS One. 2023 Oct 12;18(10):e0292248. doi: 10.1371/journal.pone.0292248 (PMC10569635; doi:10.1371/journal.pone.0292248)
Supplement: Attachment — Submitted filename: Rebutal letter.docx [file pone.0292248.s002.docx]

Journal Requirements:

When submitting your revision, we need you to address these additional requirements.

1. Please ensure that your manuscript meets PLOS ONE's **style requirements**, including those for file naming. The PLOS ONE style templates can be found at

<https://journals.plos.org/plosone/s/file?id=wjVg/PLOSOne_formatting_sample_main_body.pdf> and

<https://journals.plos.org/plosone/s/file?id=ba62/PLOSOne_formatting_sample_title_authors_affiliations.pdf>

*Corrected formatting in the manuscript and author list.*

2. Please provide additional details regarding **participant consent**. In the ethics statement in the Methods and online submission information, please ensure that you have specified (1) whether consent was informed and (2) what type you obtained (for instance, written or verbal, and if verbal, how it was documented and witnessed). If your study included minors, state whether you obtained consent from parents or guardians. If the need for consent was waived by the ethics committee, please include this information.

If you are reporting a retrospective study of medical records or archived samples, please ensure that you have discussed whether all data were fully anonymized before you accessed them and/or whether the IRB or ethics committee waived the requirement for informed consent. If patients provided informed written consent to have data from their medical records used in research, please include this information.

*Clarified in the document*

3. We note that the **grant information** you provided in the 'Funding Information' and 'Financial Disclosure' sections do not match.

When you resubmit, please ensure that you provide the correct grant numbers for the awards you received for your study in the 'Funding Information' section.

*We paid more attention to the consistency of this information.*

4. Thank you for stating the following in your Competing Interests section:

"NO"

Please complete your Competing Interests on the online submission form to state any Competing Interests. If you have no competing interests, please state **"The authors have declared that no competing interests exist.**", as detailed online in our guide for authors at http://journals.plos.org/plosone/s/submit-now

This information should be included in your cover letter; we will change the online submission form on your behalf.

*Thank you.*

5. In your Data Availability statement, you have not specified where the **minimal data set** underlying the results described in your manuscript can be found. PLOS defines a study's minimal data set as the underlying data used to reach the conclusions drawn in the manuscript and any additional data required to replicate the reported study findings in their entirety. All PLOS journals require that the minimal data set be made fully available. For more information about our data policy, please see http://journals.plos.org/plosone/s/data-availability.

Upon re-submitting your revised manuscript, please upload your study's minimal underlying data set as either Supporting Information files or to a stable, public repository and include the relevant URLs, DOIs, or accession numbers within your revised cover letter. For a list of acceptable repositories, please see [http://journals.plos.org/plosone/s/data-availability#loc-recommended-repositories](http://journals.plos.org/plosone/s/data-availability#loc-recommended-repositories). Any potentially identifying patient information must be fully anonymized.

Important: If there are ethical or legal restrictions to sharing your data publicly, please explain these restrictions in detail. Please see our guidelines for more information on what we consider unacceptable restrictions to publicly sharing data: http://journals.plos.org/plosone/s/data-availability#loc-unacceptable-data-access-restrictions. Note that it is not acceptable for the authors to be the sole named individuals responsible for ensuring data access.

We will update your Data Availability statement to reflect the information you provide in your cover letter.

*Our data is in German and the provide the answers set via email. In the figures and tables most of the data is published.*

6. One of the noted authors is a group or consortium "the CoNAN study group". In addition to naming the author group, please list the **individual authors and affiliations within this group in the acknowledgments section of your manuscript**. Please also indicate clearly a lead author for this group along with a contact email address.

*Done in the acknowledgement section*

7. Please ensure that you refer to **Figures 1 an 2** in your text as, if accepted, production will need this reference to link the reader to the figure.

*Done*.

Reviewers' comments:

Reviewer's Responses to Questions

**Comments to the Author**

1. Is the manuscript technically sound, and do the data support the conclusions?

The manuscript must describe a technically sound piece of scientific research with data that supports the conclusions. Experiments must have been conducted rigorously, with appropriate controls, replication, and sample sizes. The conclusions must be drawn appropriately based on the data presented.

Reviewer #1: Yes

Reviewer #2: Yes

2. Has the statistical analysis been performed appropriately and rigorously?

Reviewer #1: I Don't Know

Reviewer #2: Yes

3. Have the authors made all data underlying the findings in their manuscript fully available?

The [PLOS Data policy](http://www.plosone.org/static/policies.action#sharing) requires authors to make all data underlying the findings described in their manuscript fully available without restriction, with rare exception (please refer to the Data Availability Statement in the manuscript PDF file). The data should be provided as part of the manuscript or its supporting information, or deposited to a public repository. For example, in addition to summary statistics, the data points behind means, medians and variance measures should be available. If there are restrictions on publicly sharing data—e.g. participant privacy or use of data from a third party—those must be specified.

Reviewer #1: Yes

Reviewer #2: Yes

4. Is the manuscript presented in an intelligible fashion and written in standard English?

PLOS ONE does not copyedit accepted manuscripts, so the language in submitted articles must be clear, correct, and unambiguous. Any typographical or grammatical errors should be corrected at revision, so please note any specific errors here.

Reviewer #1: Yes

Reviewer #2: Yes

5. Review Comments to the Author

Please use the space provided to explain your answers to the questions above. You may also include additional comments for the author, including concerns about dual publication, research ethics, or publication ethics. (Please upload your review as an attachment if it exceeds 20,000 characters)

**We, the authors, thank the reviewers for their thoughtful and constructive comments to our manuscript. We addressed each remark extensively and revised substantially most of the discussion part to reflect on the excellent comments of both reviewers. In addition, we used the opportunity to discuss the findings and the entire manuscript with some distance from the original time of submission. As requested, we re-submit two versions: one, with (extensive) track changes; two, a clean version for better readability.**

**Again, we wish to extend our gratitude to the reviewers and their helpful remarks to improve our manuscript.**

**Reviewer #1**: An interesting study on public health risk communication from the perspective of a quarantined community.

1.1 The **importance of social media needs to be further explained** in the Introduction. Social media is a vital vehicle to provide timely health information and foster interactive communication by disseminating epidemic awareness, preventive measures, and emotional responses to the public (Budwani & Sun, 2020; Han & Xu, 2020; Liao et al., 2015; Roy et al., 2020). **It's not clear how social media was used in risk communication in the study** - it is said that inhabitants used social media to communicate with each other, so was there any impact of such communication?

*We read, discussed and included the references above and broadened the aspect of social media in the manuscript. Especially in the introduction and the discussion part, social media use and its future role in public health risk communication is mentioned more in-depth.*

1.2 It's not clear **which risk communication model/theory/framework** from the literature underpins this study. WHO is mentioned but there is no risk communication theory/model.

*We broadened the discussion about different risk communication approaches and models, but in favor of applicability for our public health audiences, we did not embark into a larger discussion on different risk communication theories. But we Included the participatory risk communication and community engagement (RCCE) approach published and applied by WHO and referenced it more precisely. We also introduced a pragmatic distinction between a traditional (sender – message – recipient model) and a modern risk communication governance approach. Our aim was to highlight important changes to risk communication practices, such as involving community leaders and to reframe social media use.*

*While the reviewer is completely right in pointing to the accuracy of explaining different theories we felt that this manuscript has a more pragmatic, practice-oriented readership that we would lose if we discussed too much theory.*

1.3 **More practical implications of the study** could be given in the Discussion, such as source credibility, which is essential for epidemic communication with the public. Research has shown that source credibility is positively related to the public's trust of health information (e.g. Kostagiolas et al., 2014; Lu et al., 2021) which, in turn, affects the public's response to the pandemic such as taking up preventive measures and sharing the information on social media (Lu et al., 2021; Vinck et al., 2019). **Areas of further research can be given in the Discussion** section too.

*The reviewer is completely right in highlighting the importance of credibility related to the public’s trust in health information, we thank him/her/them profoundly for the useful comment. We incorporated this remark and extended the discussion part.*

**Reviewer #2:** This is an interesting study that, in general, is clearly written and researched. The topic is obviously important, and will make a contribution to the related literatures as well as potentially influencing future communication strategies. There are a few clarifications needed before it is ready to publish.

2.1 38/ Quarantine is one of the most effective intervention / (should be 'interventions')

*Spelling corrected*

2.2 186-7/ The skewed age stratification of the studied case (nearly half in the village being over 60) introduces certain potential 'non-typical' aspects to this study, which raises the issue of the results also potentially being non-typical. So the description of Neustadt am Rennsteig as "a typical rural village" lacks credibility, as it presently stands, and the wording should therefore either be changed or some empirical justification given for it being "typical". (Surely, the German population as a whole doesn't have this skewed an age distribution, does it?)

*Clarified in the manuscript: rural villages have a different age distribution than cities in Germanies; with more older people. To this end, our study village is no exemption. We clarified this in the document (intro/methods and limitations).*

2.3 205-212/ Yes the sample is, at a minimal level at least, sufficiently representative, but the question remains, 'representative' of what? Of this village. So any later broad generalizations of the results will tend to look weak and are questionably justified. Be careful not to extrapolate too much from this single case (village) to broader contexts, which may or may not be similar. These limitations in potential generalizability of the results need to be acknowledged as such in the limitations section.

*Very valid point. We adjusted the tone and included more reflection in the limitation section.*

2.4 Figure 1/ This is an unnecessary graphic and should be excluded, as all of this can & should be covered in the text of the methods section. Text mention is sufficient. (This is not a problem with the other graphics.)

*Point well taken and moved to supplement.*

2.5 245, 249 etc. If this is to go in a UK- or US-english journal, the ',' format for decimals should be changed to '.' format.

*We proof read the entire document and took great care to write a consistent UK-English style.*

2.6 There is a potential problem with (likely) social desirability bias in responses to this survey. Self-report and opinion questions with regard to such a serious health crisis could be expected to reflect a greater-than-usual bias/distortion of this type. This limitation should be acknowledged in the limitations section.

*We included this aspect in the limitations part.*

Both the limitations section and the conclusion section are rather thin, as it stands. These need to be rewritten a bit more thoroughly to more completely explore implications of the research as well as its potential limitations.

*We extended both sections – discussion and limitations – significantly to reflect the helpful comments of the reviewers. Please see track changes for the changes or the clean document for the logical flow. We also rewrote the conclusion part.*
